# Global Rigidity of Two-Dimensional Bubbles

**DOI:** 10.1007/s00205-026-02262-9

**Published:** 2026-10-22

**Authors:** Lukas Niebel

**Affiliations:** Institut für Analysis und Numerik, Universität Münster, Orléans-Ring 10, 48149 Münster, Germany

## Abstract

We study bubbles, which are stationary hollow vortices with surface tension, in two dimensions. Such objects solve an overdetermined elliptic free boundary problem in an exterior domain, with an additional boundary condition involving curvature and the Neumann trace. Motivated by a conjecture of Crowdy and Wegmann, we prove that every solution prescribed by a Jordan curve is circular for $0 \le \textrm{We} \le 3$, where 3 is the first bifurcation value. This range is sharp: the bifurcation branch at $\textrm{We}=3$ contains non-circular solutions with $\textrm{We} > 3$ arbitrarily close to 3. This elliptic problem describes critical points of the sum of the perimeter and the logarithmic potential energy of compact sets. We prove an area-constrained perimeter–capacity inequality and show that, in the associated convexity-constrained variational problem, the unit disc is the unique minimiser up to translation if and only if $0 \le \textrm{We} \le 3$.

## Introduction

We study the *free boundary Euler equations* with *surface tension* in two dimensions:

$$\begin{aligned} \begin{aligned} \rho (\partial _t U + (U \cdot \nabla ) U) + \nabla P&= 0  &   \quad \text{ for } t \in \mathbb {R}, \, x \in \mathcal {B}^{\text {out}}(t) \\ \nabla \cdot U&= 0  &   \quad \text{ for } t \in \mathbb {R}, \, x \in \mathcal {B}^{\text {out}}(t) \\ -P&= \sigma \kappa  &   \quad \text{ for } t \in \mathbb {R}, \, x \in \mathcal {S}(t) \\ U \cdot n&= V_n  &   \quad \text{ for } t \in \mathbb {R}, \, x \in \mathcal {S}(t). \\ \end{aligned} \end{aligned}$$

Here *U* denotes the fluid velocity, *P* the hydrodynamic pressure, and ρ>0 the constant mass density of the fluid phase. The first two relations express momentum conservation away from the interface $\mathcal {S}(t)$ and incompressibility. Because the free interface is a moving surface carried by the fluid, its normal speed $V_n$ (i.e. $\partial _t \mathcal {S}\cdot n = V_n$) must equal the fluid’s normal velocity U·n at the interface. Motivated by bubble dynamics, we assume that the inner phase occupies a bounded and connected domain $\mathcal {B}^{\text {in}}(t)$ and is dynamically inert, with spatially constant pressure $P_{\textrm{in}}(t)$. The surrounding fluid is $\mathcal {B}^{\text {out}}(t) = \mathbb {R}^2\setminus \overline{\mathcal {B}^{\text {in}}(t)}$, so that $\mathcal {S}(t)=\partial \mathcal {B}^{\text {in}}(t)=\partial \mathcal {B}^{\text {out}}(t)$. Taking $P_{\textrm{in}}(t)$ as the pressure reference, the third relation is the *Young–Laplace law*, where σ>0 is the surface tension and κ the signed curvature. We call these objects bubbles, with a submerged air column as the model case.

We choose the signed curvature κ to be positive for convex $\mathcal {B}^{\text {in}}$, and normalise it so that κ=1 when $\mathcal {B}^{\text {in}}$ is the unit disc. The unit normal vector *n* points from the inner phase into the outer one.

### Stationary Bubbles

To find a *stationary bubble*, also called a *stationary hollow vortex solution* U=U(x) to the free boundary Euler equations with surface tension and irrotational outer flow, we have to solve an *overdetermined elliptic free boundary value problem* for the stream function $\psi = \psi (x)$ with $U = \nabla ^\perp \psi = (-\partial _2 \psi , \partial _1 \psi )$. The logarithmic asymptotics imposed below ensure that the fluid velocity vanishes at infinity. Since the stream function is defined up to an additive constant, we normalise it by requiring ψ=0 on the free boundary.

Let R>0, ρ>0, $\alpha \in \mathbb {R}$ and $\sigma \ge 0$. The task is to find a $\operatorname {C}^{1,1}$ closed Jordan curve $\mathcal {S}$ partitioning $\mathbb {R}^2$ into an interior domain $\mathcal {B}^{\text {in}}$ with $\left| \mathcal {B}^{\text {in}} \right| = \pi R^2$ and an exterior domain $\mathcal {B}^{\text {out}}$, and a solution $\psi :\mathcal {B}^{\text {out}}\rightarrow \mathbb {R}$ to

1.1 $$\begin{aligned} \begin{aligned} -\Delta \psi&= 0  &   \quad \text{ in } \mathcal {B}^{\text {out}}\\ \psi&= 0  &   \quad \text{ on } \mathcal {S}\\ \psi (x)&= \alpha \log \left| x \right| + O(1)  &   \quad \text{ as } \left| x \right| \rightarrow \infty \\ -\frac{\rho }{2}(\partial _n \psi )^2+\sigma \kappa&= \lambda  &   \quad \text{ on } \mathcal {S}\end{aligned} \end{aligned}$$

for some $\lambda \in \mathbb {R}$. The last equation is called the jump equation. By integrating over large circles anticlockwise, we observe that $2\pi \alpha$ coincides with the total circulation. To rewrite the jump condition for the pressure law, we used Bernoulli’s law for steady flows (see [34, Chapter 1.9]).

We focus on the capillary regime σ>0 with non-zero circulation $\alpha \ne 0$. The key quantity to describe the solutions of the overdetermined free boundary value problem is the *Weber number*

$$\begin{aligned} \textrm{We}= \frac{\rho \alpha ^2}{\sigma R}. \end{aligned}$$

### The Overdetermined Elliptic Free Boundary Value Problem

We nondimensionalise the problem as follows: find $\textrm{We}\ge 0$, $\lambda \in \mathbb {R}$, a $\operatorname {C}^{1,1}$ closed Jordan curve $\mathcal {S}$ partitioning $\mathbb {R}^2$ into an interior domain $\mathcal {B}^{\text {in}}$ with $\left| \mathcal {B}^{\text {in}} \right| = \pi$ and an exterior domain $\mathcal {B}^{\text {out}}$, and a solution $\psi :\mathcal {B}^{\text {out}}\rightarrow \mathbb {R}$ to

1.2 $$\begin{aligned} \begin{aligned} -\Delta \psi&= 0  &   \quad \text{ in } \mathcal {B}^{\text {out}}\\ \psi&= 0  &   \quad \text{ on } \mathcal {S}\\ \psi (x)&= \log \left| x \right| + O(1)  &   \quad \text{ as } \left| x \right| \rightarrow \infty \\ -\frac{1}{2}\textrm{We}(\partial _n \psi )^2+ \kappa&= \lambda  &   \quad \text{ on } \mathcal {S}. \end{aligned} \end{aligned}$$

Let us first explain the known solutions to (1.2). Every unit circle centred at $x_0 \in \mathbb {R}^2$ is a solution to (1.2). In fact, the function $\psi = \log \left| x-x_0 \right|$ solves the free boundary value problem and satisfies the jump condition

$$\begin{aligned} -\frac{1}{2} \textrm{We}+ 1 = \lambda . \end{aligned}$$

The first non-circular solutions were constructed by Crowdy [4], who found an explicit family of two-fold symmetric bubbles using rational conformal maps. Wegmann and Crowdy [39] subsequently generalised this construction and obtained a branch of *m*-fold symmetric bubbles for every integer m≥2. The *m*-fold branch bifurcates from the circular solution at $\textrm{We}=m+1$. Crowdy and Wegmann conjecture “that the bubble is circular for most values of circulation [equivalently of $\textrm{We}$] with the exception of an infinite series of discrete values” in [39]. Here the discrete values refer to the points at which non-circular branches bifurcate from the circular family. The main goal of this article is to prove *global rigidity* of the circular solution for $0 \le \textrm{We}\le 3$, which further supports this conjecture as $\textrm{We}=3$ is the smallest bifurcation value. We obtain the following:

#### Theorem 1.1

Let $(\mathcal {S},\psi ,\textrm{We},\lambda )$ be a solution to (1.2). If $\mathcal {S}$ is not a circle, then $\textrm{We}>3$.

No geometric assumption such as convexity, symmetry or being close to a circle is imposed.

We prove Theorem 1.1 in Section 3. We first use an exterior conformal map to rewrite the free boundary problem as a problem on the unit circle. Applying Fourier estimates to an analytic square root of the derivative of the conformal map and using the area constraint, we obtain a lower bound for $\textrm{We}$ in terms of the perimeter. The isoperimetric inequality then implies that $\textrm{We}>3$ unless $\mathcal {S}$ is a circle.

We also prove the weaker conclusion $\textrm{We}>2$ in Section 2 by combining geometric identities with identities for solutions of the elliptic PDE, such as the Pohozaev identity [27]. We believe that these ideas may be useful in higher dimensions.

The result is sharp because the two-fold Crowdy–Wegmann branch contains non-circular solutions with $\textrm{We}>3$ arbitrarily close to 3 [39]. By closely investigating the Crowdy–Wegmann branches we prove in Section 4 that both a circular and a non-circular solution exist for every $\textrm{We}\ge 10$. Numerical evidence for the remaining range $3<\textrm{We}<10$ is presented in Remark 4.3.

### Variational Bubbles

A related *variational problem* will be investigated in Section 5, where we obtain an analogous global rigidity result for its minimisers. Equation (1.2) has a variational structure analogous to non-local charged-drop models [12, 14, 15], with the logarithmic potential playing the role of the electrostatic potential.

For compact sets $E \subset \mathbb {R}^2$ of positive area and finite perimeter, we consider the functional

$$\begin{aligned} \mathcal {F}_{\textrm{We}}(E) = \textrm{We}\pi \ \mathcal {I}(E) + \mathcal {P}(E), \end{aligned}$$

with the logarithmic potential energy

$$\begin{aligned} \mathcal {I}(E) = \inf _{\begin{array}{c} \mu \text { probability measure} \\ \mu (E) = 1 \end{array}} \left\{ - \int _{\mathbb {R}^2} \int _{\mathbb {R}^2} \log \left| x-y \right| \textrm{d}\mu (y) \textrm{d}\mu (x) \right\} , \end{aligned}$$

and where $\mathcal {P}(E)$ denotes the perimeter. For smooth critical points, the correspondence with the overdetermined elliptic free boundary value problem (1.2) follows from Hadamard’s variational formula for the exterior Green function with pole at infinity; see [16]. We refer to [22] for background on logarithmic potential theory. We introduce the logarithmic capacity as ${{\,\textrm{cap}\,}}(E)=\textrm{e}^{-\mathcal {I}(E)}$.

For $\textrm{We}>0$, the variational problem

$$\begin{aligned} \inf _{\begin{array}{c} E \subset \mathbb {R}^2\\ E \text { compact}\\ \left| E \right| = \pi \end{array}} \mathcal {F}_{\textrm{We}}(E) \end{aligned}$$

is ill-posed, as can be seen by evaluating it on two discs and sending the distance between their centres to ∞. To overcome this, one could impose a further geometric constraint on *E*, such as convexity or a δ-ball condition; see [14, 15]. We restrict the minimisation problem to convex sets, since, for general Weber numbers, it is not known whether the infimum over closures of bounded domains with boundaries being rectifiable Jordan curves is attained. For the variational problem of minimising this functional over all compact and convex sets

1.3 $$\begin{aligned} \inf _{\begin{array}{c} E \subset \mathbb {R}^2\\ E \text { compact and convex} \\ \left| E \right| = \pi \end{array}} \mathcal {F}_{\textrm{We}}(E) \end{aligned}$$

global rigidity of the unit disc for small Weber numbers $\textrm{We}\le \textrm{We}_0$, for some small, quantitative but implicit constant $\textrm{We}_0$, and failure of minimality for sufficiently large $\textrm{We}$ were proved in [15]. The small-Weber-number argument depends, among other ingredients, on the sharp quantitative isoperimetric inequality due to Fusco, Maggi and Pratelli [13]. We improve this classification to the sharp range $0 \le \textrm{We}\le 3$ by proving a new inequality relating the perimeter and the logarithmic capacity of sets bounded by a Jordan curve of fixed area. Our second main result is

#### Theorem 1.2

In the class of convex and compact sets $E\subset \mathbb {R}^2$ with $\left| E \right| =\pi$, the unit disc is the unique minimiser of $\mathcal {F}_{\textrm{We}}$, up to translation, if and only if $0\le \textrm{We}\le 3$.

The key ingredient for the proof of this theorem is a new perimeter–capacity inequality

$$ \mathcal {P}(E)-2\pi \ge 2\pi \bigl (\sqrt{5{{\,\textrm{cap}\,}}(E)^2-1}-{{\,\textrm{cap}\,}}(E)-1\bigr ) $$

for compact sets $E\subset \mathbb {R}^2$ whose boundary is a rectifiable Jordan curve and which satisfy $\left| E \right| =\pi$. The area constraint makes this a refinement of the classical perimeter–capacity inequality $2\pi {{\,\textrm{cap}\,}}(E)\le \mathcal {P}(E)$, which goes back to Pólya–Schiffer and Pólya–Szegő [28–30].

The proof uses the normalised exterior conformal map of *E*. Grönwall’s area formula and a boundary representation of the perimeter express the area constraint and the perimeter in terms of a Bergman norm and a Hardy norm, respectively. Then, we take an analytic square root of the normalised derivative and apply Burbea’s sharp product inequality [2] to deduce the stated estimate.

Formally, the Euler–Lagrange equation is the overdetermined free boundary value problem (1.2). For convex sets, however, the admissible perturbations are restricted, and the last equation in (1.2) follows only on the strictly convex part of the boundary; see [21, Proposition 1]. It is therefore unclear whether minimisers of (1.3) solve the full overdetermined problem, so Theorem 1.1 does not imply Theorem 1.2.

### Literature

The two limiting cases of the problem are classical rigidity problems. For $\textrm{We}= 0$, the jump equation prescribes constant curvature, and hence $\mathcal {S}$ must be a unit circle up to translation. In the formal limit $\textrm{We}= \infty$, surface tension vanishes and (1.1) reduces to the exterior overdetermined problem with σ=0, α=1 and R=1. Serrin proved the corresponding rigidity theorem for bounded domains [35], while Reichel proved rigidity of the unit circle in the exterior setting [33, Theorem 2]. Our argument is inspired by Weinberger’s proof of Serrin’s theorem [40].

The exact families recalled above originate in Crowdy’s rational conformal maps for two-fold symmetric bubbles [4]. Wegmann and Crowdy subsequently obtained *m*-fold symmetric families of solutions to (1.2) for every integer m≥2. They numerically identified their first pinching thresholds for m=2,…,7 [39, p. 2136 and Sec. 5]. Hayford and Wang [18] later revisited these families in an equivalent electrified-droplet formulation and obtained the exact pinching thresholds for the cases m=2,3,4. The conformal mapping framework was developed further in [6]. The bifurcation values $\textrm{We}= m+1$ form a discrete sequence, whereas each nontrivial branch contains a continuum of Weber numbers. The linear analysis also indicates local rigidity of the unit circle whenever $\textrm{We}\notin \{n\in \mathbb {N}\;; \; n\ge 3\}$ as remarked in [39]. This can be made rigorous by a standard implicit-function argument, for instance using the framework of [12, 24]; since this result is not used below, we omit the details.

Crowdy, Nelson and Krishnamurthy [7] constructed rotating hollow vortices without surface tension and called them *H*-states. More generally, Crowdy and Tanveer [9] showed in several two-dimensional settings that constant-vorticity flows without surface tension and irrotational flows with surface tension reduce to the same mathematical free boundary problem. Thus, the stationary capillary bubbles considered here and the rotating *H*-states share the same free boundary profiles, although their physical equations are different. We refer to [8] for the relation between hollow vortices, capillary water waves and double quadrature domains.

The situation in the case of zero surface tension is quite different when the geometry or topology is broadened. Exceptional domains and related overdetermined problems were studied in [17, 19]. Traizet classified planar solutions and related hollow vortices to minimal surfaces in [37, 38]. Quasi-exceptional domains were studied by Eremenko and Lundberg [11]. Moreover, non-degenerate point-vortex equilibria were desingularised into translating, rotating or stationary hollow vortices in [3].

The variational problem is related to non-local models for charged liquid drops [12, 14, 15]. Crowdy [5] identified a further connection between the rational two-fold profile from [4] and static charged conducting droplets on a substrate.

We finally discuss a related three-dimensional result [24]. Close-to-spherical steady bubbles and drops with a vortex core were constructed in the three-dimensional setting. The three-dimensional axisymmetric problem with irrotational flow around the bubble is fundamentally different from its two-dimensional counterpart. For $\textrm{We}= 0$, the sphere is the unique solution. For small $\textrm{We}> 0$, the constructed solutions form the unique branch emerging from the sphere and contain all close-to-spherical solutions in the class considered there. To our knowledge, no further axisymmetric irrotational solutions with the topology of a sphere are known in this setting. It remains open whether every axisymmetric irrotational solution with sufficiently small Weber number belongs to this branch. We refer to [25] for the construction of ring-shaped solutions.

## Pohozaev and Geometric Identities: A Preliminary Rigidity Result

This section proves the following weaker version of Theorem 1.1:

### Proposition 2.1

Let $(\mathcal {S},\psi ,\textrm{We},\lambda )$ be a solution to (1.2). If $\mathcal {S}$ is not a circle, then $\textrm{We}>2$.

Fix such a solution. First, we note that $\mathcal {S}$ has to be smooth, see [20]. We recall that κ denotes the signed curvature with respect to the unit normal *n* pointing from $\mathcal {B}^{\text {in}}$ into $\mathcal {B}^{\text {out}}$. Let $L:=\mathcal {H}^1(\mathcal {S})=\mathcal {P}(\mathcal {B}^{\text {in}})>0$ and $\gamma :\mathbb {R}/(L\mathbb {Z})\rightarrow \mathcal {S}$ be an arclength parametrisation oriented anticlockwise. Set n(s):=n(γ(s)), $\kappa (s):=\kappa (\gamma (s))$, and $\tau (s):=\gamma '(s)$. Then

$$ \tau '(s)=\gamma ''(s)=-\kappa (s)n(s). $$

Writing $x=r(\cos \theta ,\sin \theta )$, for sufficiently large *r*, the exterior Fourier expansion of ψ, with $A_0,A_k,B_k\in \mathbb {R}$ for k≥1, is

$$ \psi (r,\theta ) =\log r+A_0+ \sum _{k=1}^{\infty }r^{-k} \bigl (A_k\cos (k\theta )+B_k\sin (k\theta )\bigr ). $$

Termwise differentiation gives

2.1 $$\begin{aligned} \partial _r\psi =\frac{1}{r}+O\left( \frac{1}{r^2}\right) , \qquad \left| \nabla \psi \right| ^2 =\frac{1}{r^2}+O\left( \frac{1}{r^3}\right) \quad \text {as }r\rightarrow \infty ,\ \text {uniformly in }\theta . \end{aligned}$$

We collect two simple identities. The divergence theorem, harmonicity of ψ, and (2.1) give

2.2 $$\begin{aligned} \int _{\mathcal {S}} \partial _n \psi \textrm{d}\mathcal {H}^1 = 2\pi . \end{aligned}$$

Since ψ=0 on $\mathcal {S}$, its tangential derivative vanishes there, and hence $\left| \nabla \psi \right| =\left| \partial _n\psi \right|$ on $\mathcal {S}$.

Integrating the jump condition and using the Gauß–Bonnet theorem yields

2.3 $$\begin{aligned} - \frac{\textrm{We}}{2} \int _\mathcal {S}(\partial _n \psi )^2 \textrm{d}\mathcal {H}^1 + 2\pi = \lambda \mathcal {P}(\mathcal {B}^{\text {in}}). \end{aligned}$$

Next, we use the Pohozaev identity [27] to derive a key identity. For the convenience of the reader we recall that if $u:\Omega \subset \mathbb {R}^d \rightarrow \mathbb {R}$, $d \in \mathbb {N}$, is harmonic Δu=0, then the vector field

2.4 $$\begin{aligned} X(x):=\bigl (x\cdot \nabla u(x)\bigr )\nabla u(x) -\frac{1}{2}|\nabla u(x)|^2x \end{aligned}$$

satisfies

2.5 $$\begin{aligned} \nabla \cdot X(x) =\frac{2-d}{2}|\nabla u(x)|^2. \end{aligned}$$

### Lemma 2.2

We have

$$\begin{aligned} \int _{\mathcal {S}}(x \cdot n) (\partial _n \psi )^2 \textrm{d}\mathcal {H}^1 = 2\pi . \end{aligned}$$

### Proof

Set $\Omega _{\mathscr {R}}:=B_{\mathscr {R}}\setminus \overline{\mathcal {B}^{\text {in}}}$, where $B_{\mathscr {R}}$ denotes the disc of radius $\mathscr {R}$ centred at zero and $\mathscr {R}>0$ is chosen so large that $\mathcal {B}^{\text {in}}\subset B_{\mathscr {R}}$. We consider the vector field defined in (2.4) for u:=ψ. Note that *u* is harmonic in $\mathcal {B}^{\text {out}}$, constant on $\mathcal {S}$, and has the expansion

$$ u(x)=\log \left| x \right| +O(1)\quad \text {as }\left| x \right| \rightarrow \infty . $$

Pohozaev’s identity (2.5) in dimension two simplifies to

$$ \nabla \cdot X = 0, $$

because *u* is harmonic in $\mathcal {B}^{\text {out}}$. By the divergence theorem on $\Omega _{\mathscr {R}}$ and $\nabla \cdot X=0$,

$$ 0=\int _{\Omega _{\mathscr {R}}}\nabla \cdot X \textrm{d}x =\int _{\partial \Omega _{\mathscr {R}}} X\cdot \nu \textrm{d}\mathcal {H}^1 =\int _{\partial B_{\mathscr {R}}} X\cdot \nu \textrm{d}\mathcal {H}^1 + \int _{\mathcal {S}} X\cdot \nu \textrm{d}\mathcal {H}^1, $$

where ν is the outer unit normal of $\Omega _{\mathscr {R}}$. On the outer boundary $\partial B_{\mathscr {R}}$, ν is the radial unit vector $e_r$. On the inner boundary $\mathcal {S}=\partial \mathcal {B}^{\text {in}}$, the outer normal of $\Omega _{\mathscr {R}}$ points into $\mathcal {B}^{\text {in}}$, hence ν=-n, where *n* is the unit normal of $\mathcal {S}$ pointing from $\mathcal {B}^{\text {in}}$ into $\mathcal {B}^{\text {out}}$.

On $\mathcal {S}$ we have u=0, therefore $\nabla u = (\partial _n u)n$. Using ν=-n we compute that

$$\begin{aligned} X\cdot \nu&= \big (x\cdot \nabla u\big ) \nabla u\cdot \nu - \frac{1}{2} \left| \nabla u \right| ^2 x\cdot \nu \\&= -(x\cdot n)(\partial _n u)^2 +\frac{1}{2}(x\cdot n)(\partial _n u)^2 \\&= -\frac{1}{2} (x \cdot n) (\partial _n u)^2 \end{aligned}$$

on $$\mathcal {S}$$. Therefore

$$ \int _{\mathcal {S}} X\cdot \nu \textrm{d}\mathcal {H}^1 = -\frac{1}{2}\int _{\mathcal {S}} (x \cdot n) (\partial _n u)^2 \textrm{d}\mathcal {H}^1. $$

By (2.1), on $\partial B_{\mathscr {R}}$ we have $x\cdot \nabla u=\mathscr {R}\partial _r u=1+O(1/\mathscr {R})$ and $x\cdot \nu =\mathscr {R}$. Hence,

$$ \begin{aligned} X\cdot \nu&= \big (x\cdot \nabla u\big ) \nabla u\cdot \nu - \frac{1}{2} \left| \nabla u \right| ^2 x\cdot \nu \\&= \left( 1+O\Big (\frac{1}{\mathscr {R}}\Big )\right) \Big (\frac{1}{\mathscr {R}}+O\Big (\frac{1}{\mathscr {R}^2}\Big )\Big ) - \frac{1}{2} \left( \frac{1}{\mathscr {R}^2}+O\Big (\frac{1}{\mathscr {R}^3}\Big )\right) \mathscr {R} \\&= \frac{1}{2\mathscr {R}} + O\Big (\frac{1}{\mathscr {R}^2}\Big ). \end{aligned} $$

Therefore, we have

$$ \int _{\partial B_{\mathscr {R}}} X\cdot \nu \textrm{d}\mathcal {H}^1 = \int _0^{2\pi }\Big (\frac{1}{2\mathscr {R}}+O\Big (\frac{1}{\mathscr {R}^2}\Big )\Big )\mathscr {R}\textrm{d}\theta = \pi + O(1/\mathscr {R}) \rightarrow \pi \qquad \text {as }\mathscr {R}\rightarrow \infty . $$

Collecting the two boundary contributions, we conclude

$$ 0=\lim _{\mathscr {R}\rightarrow \infty }\Big (\int _{\partial B_{\mathscr {R}}} X\cdot \nu \textrm{d}\mathcal {H}^1 + \int _{\mathcal {S}} X\cdot \nu \textrm{d}\mathcal {H}^1 \Big ) = \pi - \frac{1}{2}\int _{\mathcal {S}} (x\cdot n) (\partial _n u)^2 \textrm{d}\mathcal {H}^1. $$

□

We need the following two geometric identities:

### Lemma 2.3

We have

$$\begin{aligned} \int _{\mathcal {S}} x \cdot n \textrm{d}\mathcal {H}^1 =2\pi ,\qquad \int _{\mathcal {S}} \kappa (x \cdot n) \textrm{d}\mathcal {H}^1 = \mathcal {P}(\mathcal {B}^{\text {in}}). \end{aligned}$$

### Proof

The first formula is a simple application of the divergence theorem, using that $$\left| \mathcal {B}^{\text {in}} \right| = \pi $$. The second is the Minkowski identity for the signed curvature in two dimensions. It can be deduced from writing $$\mathcal {S}$$ as a curve γ in arc-length parametrisation oriented anticlockwise as above and integrating $$\frac{\mathrm d}{\mathrm ds}(\gamma (s) \cdot \gamma '(s)) = 1- \kappa (s) (\gamma (s) \cdot n(s))$$ over one period. Here, the normal vector *n*(*s*) is obtained by a clockwise rotation of $\gamma^{\prime }$ by π/2, and the signed curvature is defined via the Frenet formula $$\gamma ''(s) = -\kappa (s) n(s)$$. □

For every solution of (1.2), multiplying the jump condition by x·n, integrating over $\mathcal {S}$, and applying Lemmas 2.2 and 2.3 gives

2.6 $$\begin{aligned} \lambda =\frac{\mathcal {P}(\mathcal {B}^{\text {in}})}{2\pi }-\frac{\textrm{We}}{2}. \end{aligned}$$

### Proof of Proposition 2.1

If $\textrm{We}=0$, the jump condition gives $\kappa =\lambda$. Since κ is the signed curvature and $\mathcal {S}$ is a Jordan curve, the only possibility is a circle. The area constraint gives the unit circle.

Let us now consider $\textrm{We}>0$. We know that the perimeter $\mathcal {P}(\mathcal {B}^{\text {in}}) \ge 2 \pi$ by the isoperimetric inequality, since $\left| \mathcal {B}^{\text {in}} \right| = \pi$, with equality if and only if $\mathcal {S}$ is a unit circle.

Combining (2.3) and (2.6), we obtain

2.7 $$\begin{aligned} \int _{\mathcal {S}} (\partial _n \psi )^2 \textrm{d}\mathcal {H}^1 = \mathcal {P}(\mathcal {B}^{\text {in}})-\frac{\mathcal {P}(\mathcal {B}^{\text {in}})^2}{\pi \textrm{We}}+\frac{4\pi }{\textrm{We}}. \end{aligned}$$

Hence,

$$\begin{aligned} 0&\le \int _{\mathcal {S}} (\partial _n \psi )^2 \textrm{d}\mathcal {H}^1 - \frac{4\pi ^2}{\mathcal {P}(\mathcal {B}^{\text {in}})} = \mathcal {P}(\mathcal {B}^{\text {in}})-\frac{\mathcal {P}(\mathcal {B}^{\text {in}})^2}{\pi \textrm{We}}+\frac{4\pi }{\textrm{We}}- \frac{4\pi ^2}{\mathcal {P}(\mathcal {B}^{\text {in}})} \\&= \frac{1}{\textrm{We}\pi \mathcal {P}(\mathcal {B}^{\text {in}})} (\mathcal {P}(\mathcal {B}^{\text {in}})-2\pi )(\mathcal {P}(\mathcal {B}^{\text {in}})+2\pi ) (\pi \textrm{We}- \mathcal {P}(\mathcal {B}^{\text {in}})) \end{aligned}$$

by the Cauchy–Schwarz inequality and (2.2). If $\mathcal {S}$ is not a unit circle, then $\mathcal {P}(\mathcal {B}^{\text {in}})>2\pi$ and we deduce that $\textrm{We}> 2$. □

### Remark 2.4

The estimate uses only the sharp constants in the isoperimetric inequality and the Cauchy–Schwarz inequality.

## Global Rigidity of the Unit Circle for $\textrm{We}\le 3$

We identify $\mathbb {R}^2$ with $\mathbb {C}$ and set

$$ \mathbb {D}:=\{z\in \mathbb {C}\; ; \; \left| z \right| <1\}. $$

We first fix the normalisation of the exterior Riemann map used in this section and in Sections 4 and 5. Let $E\subset \mathbb {C}$ be the closure of a bounded Jordan domain. By the exterior Riemann mapping theorem, there is a unique conformal map fixing infinity whose leading coefficient at infinity is positive. We denote this map by Φ and write its Laurent expansion at infinity as

3.1 $$\begin{aligned} \begin{aligned} \Phi :(\mathbb {C}\cup \{\infty \})\setminus \overline{\mathbb {D}} \longrightarrow (\mathbb {C}\cup \{\infty \})\setminus E,\\ \Phi (\infty )=\infty , \qquad \Phi '(\infty ):= \lim _{\zeta \rightarrow \infty }\frac{\Phi (\zeta )}{\zeta }=c>0,\\ \Phi (\zeta ) =c\left( \zeta +\beta _0+\sum _{n=1}^{\infty }\beta _n\zeta ^{-n} \right) , \qquad \left| \zeta \right| >1. \end{aligned} \end{aligned}$$

We call Φ the normalised exterior conformal map of *E*. The coefficients $\beta _0,\beta _1,\ldots \in \mathbb {C}$ are the corresponding normalised Laurent coefficients. We next record Grönwall’s area formula for the normalised exterior conformal map.

### Lemma 3.1

Let $E\subset \mathbb {C}$ be compact with boundary given by a rectifiable Jordan curve, and let Φ, *c*, and $(\beta _n)_{n\ge 0}$ be as in (3.1). Then $c=\Phi '(\infty )={{\,\textrm{cap}\,}}(E)$ and

3.2 $$\begin{aligned} \left| E \right| =\pi c^2\left( 1-\sum _{n=1}^{\infty }n\left| \beta _n \right| ^2 \right) . \end{aligned}$$

### Proof

The normalisation of the exterior Riemann map gives $$c={{\,\textrm{cap}\,}}(E)$$; see [32, Thm. 5.2.3] and [23, Sec. 2, p. 691]. We apply the area computation in the proof of Grönwall’s area theorem [36, Thm. 1.2.1] to $$G:=c^{-1}\Phi $$, for which $G^{\prime }\left(\infty \right)=1$, and then rescale by *c* to obtain (3.2). □

### Proof of Theorem 1.1

Let us assume that $(\mathcal {S},\psi ,\textrm{We},\lambda )$ solves (1.2) and that $\mathcal {S}$ is not a circle. Again, by [20] the curve $\mathcal {S}$ is smooth.

Following the exterior conformal formulation of [39, (4)–(7)], let Φ be the normalised exterior conformal map in (3.1) associated with $$E=\overline{\mathcal {B}^{\text {in}}}$$. By Lemma 3.1, its leading coefficient is $$c={{\,\textrm{cap}\,}}(\overline{\mathcal {B}^{\text {in}}})$$. Applying the Kellogg–Warschawski theorem to the exterior conformal map, after inversion ζ=1/z, we find that Φ extends smoothly to $$\left| \zeta \right| =1$$ and that $\Phi^{\prime }$ does not vanish there [31, Thms. 3.5 and 3.6, pp. 48–49].

The function $\psi \circ \Phi -\log \left| \zeta \right|$ is bounded and harmonic for $\left| \zeta \right| >1$ and has zero boundary values on $\left| \zeta \right| =1$. After the inversion ζ=1/z, the resulting bounded harmonic function has a removable singularity at the origin. The maximum principle, followed by continuity to the boundary, gives

$$ \psi (\Phi (\zeta ))=\log \left| \zeta \right| \qquad \text {for }\left| \zeta \right| \ge 1. $$

Therefore, on $$\zeta =\textrm{e}^{\textrm{i}t}$$, $$t\in [0,2\pi )$$,

3.3 $$\begin{aligned} \textrm{d}\mathcal {H}^1\bigl (\Phi (\textrm{e}^{\textrm{i}t})\bigr )=\left| \Phi '(\textrm{e}^{\textrm{i}t}) \right| \textrm{d}t, \qquad [\partial _n\psi ](\Phi (\textrm{e}^{\textrm{i}t}))=\left| \Phi '(\textrm{e}^{\textrm{i}t}) \right| ^{-1}. \end{aligned}$$

We set

$$ G(z):=\Phi '(1/z)\quad \text {for }0<\left| z \right| <1, \qquad G(0):=c. $$

The function *G* is analytic and zero-free in the disc and extends smoothly and without zeros to the unit circle. Since the disc is simply connected, it has an analytic logarithm *L*, which we normalise by L(0)=logc; see [1, Ch. 4, Sec. 4.4, Cor. 2, p. 142]. Let $\Lambda =(-\partial _t^2)^{1/2}$ on 2π-periodic functions, so that

3.4 $$\begin{aligned} \Lambda \textrm{e}^{\textrm{i}k t}=\left| k \right| \textrm{e}^{\textrm{i}k t}, \qquad k \in \mathbb {Z}. \end{aligned}$$

We set $\gamma (t):=\Phi (\textrm{e}^{\textrm{i}t})$, which parametrises $\mathcal {S}$ anticlockwise, the signed curvature is

$$ \kappa (\gamma (t)) = \frac{1}{\left| \gamma '(t) \right| } \operatorname {Im}\left( \frac{\gamma ''(t)}{\gamma '(t)}\right) . $$

Since

$$ \frac{\gamma ''(t)}{\gamma '(t)} = \textrm{i}\left( 1+\textrm{e}^{\textrm{i}t}\frac{\Phi ''(\textrm{e}^{\textrm{i}t})}{\Phi '(\textrm{e}^{\textrm{i}t})} \right) $$

and $G(\textrm{e}^{-\textrm{i}t})=\Phi '(\textrm{e}^{\textrm{i}t})$, that analyticity of *L* and (3.4) give

$$ \Lambda \log \left| \Phi '(\textrm{e}^{\textrm{i}t}) \right| =-\operatorname {Re}\left( \textrm{e}^{\textrm{i}t}\frac{\Phi ''(\textrm{e}^{\textrm{i}t})}{\Phi '(\textrm{e}^{\textrm{i}t})}\right) . $$

Consequently, the curvature of the conformal parametrisation is

3.5 $$\begin{aligned} \kappa (\Phi (\textrm{e}^{\textrm{i}t})) = \frac{1-\Lambda \log \left| \Phi '(\textrm{e}^{\textrm{i}t}) \right| }{\left| \Phi '(\textrm{e}^{\textrm{i}t}) \right| }. \end{aligned}$$

Substituting (3.3) and (3.5) into the jump condition in (1.2), and using (2.6), we obtain

$$ \frac{1-\Lambda \log \left| \Phi '(\textrm{e}^{\textrm{i}t}) \right| }{\left| \Phi '(\textrm{e}^{\textrm{i}t}) \right| } - \frac{\textrm{We}}{2}\left| \Phi '(\textrm{e}^{\textrm{i}t}) \right| ^{-2} = \frac{\mathcal {P}(\mathcal {B}^{\text {in}})}{2\pi }-\frac{\textrm{We}}{2}. $$

We set

$$ D_\Phi :=\frac{1}{2\pi }\int _0^{2\pi }\left| \Phi '(\textrm{e}^{\textrm{i}t}) \right| ^2\textrm{d}t-1. $$

Multiplying the preceding identity by $\left| \Phi '(\textrm{e}^{\textrm{i}t}) \right| ^2$ and averaging in *t*, using the first equation in (3.3), gives

3.6 $$\begin{aligned}&\frac{1}{2\pi }\int _0^{2\pi } \left| \Phi '(\textrm{e}^{\textrm{i}t}) \right| \,\Lambda \log \left| \Phi '(\textrm{e}^{\textrm{i}t}) \right| \textrm{d}t \\&= \left( \frac{\textrm{We}}{2}-\frac{\mathcal {P}(\mathcal {B}^{\text {in}})}{2\pi }\right) D_\Phi . \nonumber \end{aligned}$$

We now use the analytic square-root representation of the boundary length. Set F:=exp(L/2), so that

$$ F(z)^2=G(z), \qquad F(0)=c^{1/2}, \qquad F(z)=\sum _{n=0}^{\infty }c_nz^n, \qquad z\in \mathbb {D}. $$

From (3.1),

$$ G(z) =c\left( 1-\sum _{m=1}^{\infty }m\beta _m z^{m+1} \right) , $$

so the coefficient of *z* in $F(z)^2$ vanishes. Since $c_0^2=c>0$, we have $c_1=0$. The functions *F* and L/2=logF have smooth boundary traces. Moreover, $G(\textrm{e}^{-\textrm{i}t})=\Phi '(\textrm{e}^{\textrm{i}t})$ gives

$$ \left| \Phi '(\textrm{e}^{\textrm{i}t}) \right| =\left| F(\textrm{e}^{-\textrm{i}t}) \right| ^2, \qquad \log \left| \Phi '(\textrm{e}^{\textrm{i}t}) \right| =2\operatorname {Re}\log F(\textrm{e}^{-\textrm{i}t}). $$

Changing variables once and using Parseval’s identity therefore yields

3.7 $$\begin{aligned} \frac{\mathcal {P}(\mathcal {B}^{\text {in}})}{2\pi } = \frac{1}{2\pi }\int _0^{2\pi }\left| \Phi '(\textrm{e}^{\textrm{i}t}) \right| \textrm{d}t = \frac{1}{2\pi }\int _0^{2\pi }\left| F(\textrm{e}^{\textrm{i}t}) \right| ^2\textrm{d}t = \sum _{n=0}^{\infty }\left| c_n \right| ^2. \end{aligned}$$

The same boundary relations give

3.8 $$\begin{aligned} \frac{1}{2\pi }\int _0^{2\pi } \left| \Phi '(\textrm{e}^{\textrm{i}t}) \right| \,\Lambda \log \left| \Phi '(\textrm{e}^{\textrm{i}t}) \right| \textrm{d}t&= \frac{1}{2\pi }\int _0^{2\pi } \left| F(\textrm{e}^{\textrm{i}t}) \right| ^2\,2\operatorname {Re}\!\left( \frac{\textrm{e}^{\textrm{i}t}F'(\textrm{e}^{\textrm{i}t})}{F(\textrm{e}^{\textrm{i}t})}\right) \textrm{d}t \nonumber \\&= 2\operatorname {Re}\left( \frac{1}{2\pi }\int _0^{2\pi } \textrm{e}^{\textrm{i}t}F'(\textrm{e}^{\textrm{i}t})\overline{F(\textrm{e}^{\textrm{i}t})}\textrm{d}t \right) \nonumber \\&= 2\sum _{n=0}^{\infty }n\left| c_n \right| ^2. \end{aligned}$$

By Lemma 3.1 and $\left| \mathcal {B}^{\text {in}} \right| =\pi$, we have

$$ 1 =c^2\left( 1-\sum _{m=1}^{\infty }m\left| \beta _m \right| ^2 \right) . $$

On the other hand, Parseval’s identity gives

$$ \frac{1}{2\pi }\int _0^{2\pi }\left| \Phi '(\textrm{e}^{\textrm{i}t}) \right| ^2\textrm{d}t =c^2\left( 1+\sum _{m=1}^{\infty }m^2\left| \beta _m \right| ^2 \right) . $$

Since the coefficient of $z^k$ in $F(z)^2=G(z)$ is $\sum _{j=0}^{k}c_jc_{k-j}$, comparison with the preceding expansion of *G* gives, for k≥2,

$$ \sum _{j=0}^{k}c_jc_{k-j} =-c(k-1)\beta _{k-1}. $$

Consequently, subtracting the preceding area identity from the Parseval identity gives

3.9 $$\begin{aligned} D_\Phi = \sum _{k=2}^{\infty }\frac{k}{k-1} \left| \sum _{j=0}^{k}c_jc_{k-j} \right| ^2. \end{aligned}$$

In particular, $$D_\Phi $$ vanishes if and only if $\Phi^{\prime }$ is constant. By the area normalisation this is equivalent to $$\mathcal {S}$$ being a unit circle. Since we assumed that $$\mathcal {S}$$ is not a circle we have $$D_\Phi >0$$.

We now estimate the right-hand side in (3.9). As $c_1=0$, for every k≥2 the sum $\sum _{j=0}^{k}c_jc_{k-j}$ has at most *k* non-zero summands. Therefore Cauchy’s inequality, k/(k-1)≤2, and (3.7) imply

3.10 $$\begin{aligned} D_\Phi&\le 2\sum _{k=2}^{\infty }k\sum _{j=0}^{k}\left| c_j \right| ^2\left| c_{k-j} \right| ^2 = 2\sum _{i,j=0}^{\infty }(i+j)\left| c_i \right| ^2\left| c_j \right| ^2 \nonumber \\&=4\frac{\mathcal {P}(\mathcal {B}^{\text {in}})}{2\pi } \sum _{j=0}^{\infty }j\left| c_j \right| ^2. \end{aligned}$$

Now (3.6) and (3.8) imply

$$ 2\sum _{n=0}^{\infty }n\left| c_n \right| ^2 = \left( \frac{\textrm{We}}{2}-\frac{\mathcal {P}(\mathcal {B}^{\text {in}})}{2\pi }\right) D_\Phi . $$

We may divide by $D_\Phi$ and obtain

$$ \frac{\textrm{We}}{2} = \frac{\mathcal {P}(\mathcal {B}^{\text {in}})}{2\pi } + \frac{2\sum _{n=0}^{\infty }n\left| c_n \right| ^2}{D_\Phi }. $$

Since $$D_\Phi >0$$, (3.10) gives

$$ \frac{2\sum _{n=0}^{\infty }n\left| c_n \right| ^2}{D_\Phi } \ge \frac{1}{2\frac{\mathcal {P}(\mathcal {B}^{\text {in}})}{2\pi }}. $$

Consequently,

3.11 $$\begin{aligned} \frac{\textrm{We}}{2} \ge \frac{\mathcal {P}(\mathcal {B}^{\text {in}})}{2\pi } + \frac{1}{2\frac{\mathcal {P}(\mathcal {B}^{\text {in}})}{2\pi }}. \end{aligned}$$

Since $\left| \mathcal {B}^{\text {in}} \right| =\pi$ and $\mathcal {S}$ is not a unit circle, the isoperimetric inequality gives $\mathcal {P}(\mathcal {B}^{\text {in}})>2\pi$. Hence

$$ \frac{\mathcal {P}(\mathcal {B}^{\text {in}})}{2\pi } + \frac{1}{2\frac{\mathcal {P}(\mathcal {B}^{\text {in}})}{2\pi }} - \frac{3}{2} = \frac{\left( \frac{\mathcal {P}(\mathcal {B}^{\text {in}})}{2\pi }-1\right) \left( 2\frac{\mathcal {P}(\mathcal {B}^{\text {in}})}{2\pi }-1\right) }{2\frac{\mathcal {P}(\mathcal {B}^{\text {in}})}{2\pi }} > 0. $$

Thus (3.11) implies $\textrm{We}>3$. This proves the theorem. □

## The Crowdy–Wegmann Branches

In this section, we present the non-circular solutions constructed by Crowdy and Wegmann [4, 39] and calculate the parameter at which their boundary curves first cease to be Jordan curves. We use the same conformal formulation as in the proof of Theorem 1.1.

A bubble with boundary prescribed by a Jordan curve determines a normalised exterior conformal map Φ as in (3.1), and its potential is characterised by $$\psi (\Phi (\zeta ))=\log \left| \zeta \right| $$ for $$\left| \zeta \right| >1$$. Conversely, suppose that Φ is holomorphic and univalent in $$\mathbb {C}\setminus \overline{\mathbb {D}}$$, has the normalisation at infinity from (3.1), and extends $$\operatorname {C}^2$$-smoothly to the unit circle, with $$\Phi '\ne 0$$ there and an injective boundary trace. Then $$\Gamma :=\Phi (\partial \mathbb {D})$$ is a Jordan curve, and the same relation defines a potential satisfying the first three conditions in (1.2). After a dilation which fixes the enclosed area, (3.3) and (3.5) express the remaining jump condition in (1.2) entirely in terms of the boundary values of $\Phi^{\prime }$. Thus constructing a bubble reduces to finding such a conformal map for which this form of the jump condition holds.

Crowdy [4] found the two-fold symmetric solution family, and Wegmann and Crowdy [39] subsequently generalised it to *m*-fold symmetric maps for every integer m≥2. Up to translation and rotation, we may take the parameter *a* of [39] to be nonnegative and set $b:=a^m$. The derivative $\Phi _{m,b}'$ is the function $h_m$ in their equation (26), and $\Phi _{m,b}$ is the primitive $f_m$ in their equation (31). Their exterior map, normalised at infinity as in (3.1) but not yet rescaled to enclose area π, and its derivative are

4.1 $$\begin{aligned} \Phi _{m,b}(\zeta ) = \zeta + \frac{4mb\zeta }{(m-1)(\zeta ^m+(m-1)b)}, \qquad \Phi _{m,b}'(\zeta ) = \left( \frac{\zeta ^m-(m+1)b}{\zeta ^m+(m-1)b} \right) ^2. \end{aligned}$$

For fixed *m* and *b*, we set $\Gamma _{m,b}:=\Phi _{m,b}(\partial \mathbb {D})$. We call *b* admissible if $\Phi _{m,b}$ is univalent in $\mathbb {C}\setminus \overline{\mathbb {D}}$ and its boundary trace is injective. Then $\Gamma _{m,b}$ is a Jordan curve. As *b* increases, we are therefore interested in the first parameter at which two distinct points of $\partial \mathbb {D}$ have the same image. At this parameter, two distinct parts of $\Gamma _{m,b}$ meet, the boundary trace ceases to be injective, and the curve is no longer a Jordan curve. Thus the first such parameter is the endpoint of the conformal branch in the class of Jordan curves.

For every admissible parameter, [39, (35)] gives the area enclosed by $\Gamma _{m,b}$ as $\pi \mathfrak a_m(b)$, where

4.2 $$\begin{aligned} \mathfrak a_m(b) := 1- \frac{16m^2b^2}{m-1} \frac{1+(m-1)b^2}{(1-(m-1)^2b^2)^2}. \end{aligned}$$

Thus $\mathfrak a_m(b)>0$ for every such parameter. Taking the positive square root, we set

$$ \widehat{\Phi }_{m,b} :=\mathfrak a_m(b)^{-1/2}\Phi _{m,b}, \qquad \mathcal {S}_{m,b}:=\widehat{\Phi }_{m,b}(\partial \mathbb {D}). $$

The curve $\mathcal {S}_{m,b}$ encloses area π. On its exterior, we define the corresponding potential by

$$ \psi _{m,b}(\widehat{\Phi }_{m,b}(\zeta )) =\log \left| \zeta \right| , \qquad \left| \zeta \right| >1. $$

Combining [39, (29),(30),(35)] with our normalisation conventions, we find a constant $\lambda _{m,b}$ such that $(\mathcal {S}_{m,b},\psi _{m,b},\textrm{We}_m(b),\lambda _{m,b})$ solves (1.2), where

4.3 $$\begin{aligned} \textrm{We}_m(b) = (m+1) \frac{1-(m+1)^2b^2}{1+(m-1)(m+1)b^2} \,\mathfrak a_m(b)^{-1/2}. \end{aligned}$$

The coefficient of $\left| f' \right| ^{-1}$ in the boundary equation of [39] is $\textrm{We}/2$ in our normalisation. At b=0, the map $\Phi _{m,0}$ is the identity and $\textrm{We}_m(0)=m+1$. Hence the *m*-th branch emanates from the circular family at the bifurcation value $\textrm{We}=m+1$. Expanding (4.3), in agreement with [39, (39)], shows that the branches with m=2,3,4 initially move towards larger Weber numbers, whereas those with m≥5 initially move towards smaller Weber numbers. In particular,

$$ \textrm{We}_2(b)=3+60b^2+O(b^4), \qquad b\downarrow 0, $$

and hence the non-circular branch emanating from $\textrm{We}=3$ immediately lies in the range $\textrm{We}>3$.

Wegmann and Crowdy numerically identified these parameters for m=2,…,7 in terms of *a* and observed that the critical curves undergo an *m*-fold pinching [39, p. 2136 and Sec. 5]. The exact thresholds for m=2,3,4 were subsequently obtained by Hayford and Wang in an equivalent formulation [18]. We determine the corresponding thresholds explicitly for every m≥2. For each integer k≥0, let $U_k$ denote the Chebyshev polynomial of the second kind and set

4.4 $$\begin{aligned} D_m(x) := -(m-1)U_m(x)+\frac{(m+1)^2}{m-1}U_{m-2}(x), \qquad M_m:=\max _{-1\le x\le 1}\left| D_m(x) \right| . \end{aligned}$$

Using $U_k'(1)=k(k+1)(k+2)/3$, we find that

$$ D_m(1)=2(m+1), \qquad D_m(-1)=2(-1)^m(m+1), \qquad D_m'(1)=-\frac{2m^2(m+1)}{3}<0. $$

Thus $D_m(x)>D_m(1)$ for x<1 sufficiently close to 1. Consequently, $M_m>2(m+1)$, and we may define that

4.5 $$\begin{aligned} b_m^* := \frac{2}{M_m+\sqrt{M_m^2-4(m+1)^2}}. \end{aligned}$$

In particular, $$0<b_m^*<1/(m+1)$$.

### Lemma 4.1

For every m≥2 and $0\le b<b_m^*$, the map $\Phi _{m,b}$ is univalent in $\mathbb {C}\setminus \overline{\mathbb {D}}$ and its boundary trace is injective. At $b=b_m^*$, two distinct points of the unit circle have the same image under $\Phi _{m,b}$.

### Proof

Let $z,w\in \partial \mathbb {D}$ with z≠w. We write that

$$ z=\textrm{e}^{\textrm{i}(\varphi +\theta )}, \qquad w=\textrm{e}^{\textrm{i}(\varphi -\theta )}, \qquad \varphi \in \mathbb {R}, \qquad 0<\theta <\pi . $$

For every integer k≥0, we have

$$ \sum _{j=0}^k z^{k-j}w^j = \textrm{e}^{\textrm{i}k\varphi }U_k(\cos \theta ). $$

Applying this to the difference of the two rational functions in (4.1), we obtain the factorisation

4.6 $$\begin{aligned} \Phi _{m,b}(z)-\Phi _{m,b}(w) = \frac{(z-w)\left( \textrm{e}^{2\textrm{i}m\varphi } -bD_m(\cos \theta )\textrm{e}^{\textrm{i}m\varphi } +(m+1)^2b^2 \right) }{ (z^m+(m-1)b)(w^m+(m-1)b) }. \end{aligned}$$

Suppose that 0<b<1/(m+1) and that the last factor in the numerator vanishes. Setting $\omega :=\textrm{e}^{\textrm{i}m\varphi }$, dividing (4.6) by ω, and taking imaginary parts gives

$$ \bigl (1-(m+1)^2b^2\bigr )\sin (m\varphi )=0. $$

Thus $\omega \in \{-1,1\}$, and the real part of (4.6) yields

4.7 $$\begin{aligned} \left| D_m(\cos \theta ) \right| = \frac{1}{b}+(m+1)^2b. \end{aligned}$$

Conversely, if (4.7) holds for some $0<\theta <\pi$, we can choose φ such that $\textrm{e}^{\textrm{i}m\varphi }$ has the sign of $D_m(\cos \theta )$. Then (4.6) shows that two distinct boundary points have the same image under $\Phi _{m,b}$.

The function $b\mapsto b^{-1}+(m+1)^2b$ is strictly decreasing on (0,1/(m+1)). Moreover, (4.5) is its unique solution of

$$ \frac{1}{b}+(m+1)^2b=M_m $$

in this interval. Hence (4.7) cannot hold for $0<b<b_m^*$. Since $M_m>2(m+1)$, every maximiser of $\left| D_m \right|$ lies in (-1,1). Choosing such a maximiser and applying the converse implication above, we obtain distinct points $z,w\in \partial \mathbb {D}$ such that

$$ \Phi _{m,b_m^*}(z)=\Phi _{m,b_m^*}(w). $$

Finally, the formulae in (4.1) show that $\Phi _{m,b}$ has neither a pole nor a critical point in $\mathbb {C}\setminus \mathbb {D}$ for $0\le b<b_m^*$. Its boundary trace is injective by the preceding argument, and $\Phi _{m,b}(\zeta )=\zeta +O(\left| \zeta \right| ^{1-m})$ as $\left| \zeta \right| \rightarrow \infty$. The argument principle [1, Ch. 4, Sec. 5.2] now shows that $\Phi _{m,b}$ maps $\mathbb {C}\setminus \overline{\mathbb {D}}$ conformally onto the unbounded component of the complement of its boundary curve. This proves the claim. □

### Proposition 4.2

For every $\textrm{We}\ge 10$, there exists a non-circular Crowdy–Wegmann bubble of area π which solves (1.2) with Weber number $\textrm{We}$.

### Proof

For $0<\theta <\pi$, the trigonometric representation of the Chebyshev polynomials gives

4.8 $$\begin{aligned} D_m(\cos \theta ) = \frac{2}{m-1}\left( 2m\cot \theta \sin (m\theta ) -(m^2+1)\cos (m\theta ) \right) . \end{aligned}$$

The values at θ=0 and $\theta =\pi$ are understood by continuous extension. We claim that

$$ M_m\le 3(m+1) \qquad m\ge 4. $$

At the endpoints, the identities above give

$$ \left| D_m(1) \right| = \left| D_m(-1) \right| = 2(m+1) \le 3(m+1). $$

Since $M_m>2(m+1)$, the maximum is attained at some $\theta \in (0,\pi )$ with

$$ \left| D_m(\cos \theta ) \right| =M_m. $$

Differentiating (4.8) at this maximiser shows that the desired estimate is equivalent, with $y=\sin ^2\theta$ and $h=m^2-1$, to

$$ 5h^2y^2-(16h+36)y+20\ge 0. $$

The discriminant of this quadratic polynomial is -144(h-9)(h+1)<0 for m≥4, while its leading coefficient is positive. This proves the claim. Recalling (4.5), we deduce that

$$ b_m^* \ge \frac{3-\sqrt{5}}{2(m+1)} >\frac{1}{3(m+1)}. $$

Let $m \in \mathbb {N}$ with m≥10 and choose $b_0:=1/(3(m+1))$. Lemma 4.1 shows that $\Phi _{m,b}$ is univalent for $0\le b\le b_0$. Substituting $b_0$ into (4.3), we obtain

4.9 $$\begin{aligned} \bigl [\textrm{We}_m(b_0)\bigr ]^2 = \frac{ 16(m-1)(m+1)^2(2m+1)^2(m+2)^2 }{ (5m+4)^2(4m^3-m^2-8m-4) } <m^2. \end{aligned}$$

The last inequality is equivalent to Q(m)>0, where we set

$$ Q(m) := 36m^7-249m^6-960m^5-900m^4 +176m^3+720m^2+384m+64. $$

Writing m=n+10 with an integer n≥0, every coefficient of Q(n+10) is strictly positive. Hence Q(m)>0 for m≥10. Moreover, $0<b_0<1/(m+1)$, and therefore (4.3) gives $\textrm{We}_m(b_0)>0$. In view of (4.9), we conclude that $\textrm{We}_m(b_0)<m$.

As noted above, $\textrm{We}_m(0)=m+1$. Hence continuity yields every value in [m,m+1) for some $0<b<b_m^*$. Since $\bigcup _{m\ge 10}[m,m+1)=[10,\infty )$, the proposition follows. □

### Remark 4.3

A Mathematica investigation of the branch ranges in $3<\textrm{We}<10$ suggests that the non-circular Crowdy–Wegmann branches cover all Weber numbers $\textrm{We}>3$, except possibly in two intervals; their endpoint pairs are estimated to be near

$$ 8.18307625\ldots ,\ 8.37519009\ldots \qquad \text {and}\qquad 9,\ 9.06929546\ldots . $$

## On a Related Variational Problem

We now turn to the variational problem (1.3).

### Minimality of the Disc for $0 \le \textrm{We}\le 3$

For $1\le p<\infty$ and an analytic function *u* on $\mathbb {D}$, we use the Hardy norm

$$ \left\| u \right\| _{\mathcal {H}^p}^p := \sup _{0<r<1}\frac{1}{2\pi }\int _0^{2\pi }\left| u(r\textrm{e}^{\textrm{i}t}) \right| ^p\textrm{d}t. $$

We also write that

$$ \left\| u \right\| _{A^2}^2:=\frac{1}{\pi }\int _{\mathbb {D}}\left| u(z) \right| ^2\textrm{d}A(z) $$

for the Bergman norm, where $\textrm{d}A$ denotes planar Lebesgue measure. We denote by $\mathcal {H}^p(\mathbb {D})$ and $A^2(\mathbb {D})$ the corresponding spaces of analytic functions with finite norm. For $u\in \mathcal {H}^p(\mathbb {D})$, we write that

$$ u^*(\textrm{e}^{\textrm{i}t}) := \lim _{r\uparrow 1}u(r\textrm{e}^{\textrm{i}t}) $$

for its radial boundary value, which exists for Lebesgue-almost every $t\in [0,2\pi )$. For further background on Hardy and Bergman spaces, see [26, Ch. 5 and Sec. 8.1].

The key tool for Theorem 1.2 is the following area-constrained perimeter–capacity estimate, whose proof uses three auxiliary lemmas.

#### Proposition 5.1

Let $E\subset \mathbb {R}^2\simeq \mathbb {C}$ be compact with boundary given by a rectifiable Jordan curve, and suppose that $\left| E \right| =\pi$. Then

$$ \mathcal {P}(E)-2\pi \ge 2\pi \bigl (\sqrt{5{{\,\textrm{cap}\,}}(E)^2-1}-{{\,\textrm{cap}\,}}(E)-1\bigr ). $$

Moreover, equality holds if and only if *E* is a translate of the unit disc.

The first one is the classical area–capacity inequality; see [32, Thm. 5.3.5].

#### Lemma 5.2

Let *E* satisfy the assumptions of Proposition 5.1. Then

$$ {{\,\textrm{cap}\,}}(E)\ge 1. $$

#### Proof

Let Φ, *c*, and $(\beta _n)_{n\ge 0}$ be as in (3.1). By Lemma 3.1, $c={{\,\textrm{cap}\,}}(E)$. Since $\left| E \right| =\pi$, equation (3.2) gives

$$ 1 =c^2\left( 1-\sum _{n=1}^{\infty }n\left| \beta _n \right| ^2 \right) \le c^2. $$

□

Next, we express the perimeter as a Hardy norm.

#### Lemma 5.3

Let *E* satisfy the assumptions of Proposition 5.1, and suppose that $0\in \operatorname {int}(E)$. Let Φ be the corresponding normalised exterior conformal map, with *c* and $(\beta _n)_{n\ge 0}$ as in (3.1). Define that

$$ q(z):=\sum _{n=1}^{\infty }n\beta _nz^{n+1}, \qquad z\in \mathbb {D}. $$

Then

$$ q(0)=q'(0)=0, \qquad 1-q(z)\ne 0\quad \text {for all }z\in \mathbb {D}. $$

Here and below, $$q/z^2$$ denotes its analytic continuation at the origin, with value $q^{\prime \prime }\left(0\right)/2$ there. The area and perimeter satisfy that

5.1 $$\begin{aligned} \left\| \frac{q}{z^2} \right\| _{A^2}^2 = \sum _{n=1}^{\infty }n\left| \beta _n \right| ^2 = 1-\frac{\left| E \right| }{\pi c^2}. \end{aligned}$$

5.2 $$\begin{aligned} \frac{\mathcal {P}(E)}{2\pi c}=\left\| 1-q \right\| _{\mathcal {H}^1}. \end{aligned}$$

#### Proof

The coefficient formula for the Bergman norm [26, proof of Thm. 5.3.4, p. 77], together with (3.2), gives (5.1). By Carathéodory’s theorem, Φ extends continuously and one-to-one to $\partial \mathbb {D}$; see [1, Ch. 6, Sec. 1.2]. For $0<\left| z \right| <1$, set that

$$ f(z):=\frac{1}{\Phi (1/z)}, \qquad h(z):=z\Phi (1/z), $$

and set that f(0):=0 and h(0):=c. The Laurent expansion of Φ shows that these are the removable analytic extensions to $\mathbb {D}$. Inversion $z \mapsto \frac{1}{z}$ is bi-Lipschitz in a neighbourhood of ∂E. Hence *f* maps $\mathbb {D}$ conformally onto a bounded Jordan domain with rectifiable boundary. By the rectifiable-boundary theorem [10, Thm. 3.12, p. 44], we have $f'\in \mathcal {H}^1(\mathbb {D})$. The same boundary extension shows that *h* is bounded. Therefore, for $0<\left| z \right| <1$,

$$ 1-q(z)=\frac{1}{c}\Phi '(1/z)=\frac{1}{c} h(z)^2f'(z). $$

The identity extends analytically across 0; since *h* is bounded and $f'\in \mathcal {H}^1(\mathbb {D})$, it follows that $1-q\in \mathcal {H}^1(\mathbb {D})$. The boundary map $t\mapsto f(\textrm{e}^{\textrm{i}t})$ is absolutely continuous and satisfies

$$ \frac{\mathrm d}{\mathrm dt}f(\textrm{e}^{\textrm{i}t})=\textrm{i}\textrm{e}^{\textrm{i}t}(f')^*(\textrm{e}^{\textrm{i}t}) $$

for almost every *t*; see [10, Thm. 3.11, p. 42]. Since *f* is bounded away from zero on $\partial \mathbb {D}$, the boundary parametrisation $\gamma (t):=\Phi (\textrm{e}^{\textrm{i}t})=1/f(\textrm{e}^{-\textrm{i}t})$ is absolutely continuous and satisfies

$$ \left| \gamma '(t) \right| = c\left| (1-q)^*(\textrm{e}^{-\textrm{i}t}) \right| \qquad \text {for almost every }t\in [0,2\pi ]. $$

The boundary-value identity for the Hardy norm [26, Thm. 5.1.8] now gives $\mathcal {P}(E)=2\pi c\left\| 1-q \right\| _{\mathcal {H}^1}$. Finally, 1-q(0)=1, while for $0<\left| z \right| <1$ it is nonzero because $\Phi '(1/z)\ne 0$. This proves the claim. □

It remains to relate the Hardy norm in (5.2) to the Bergman norm in (5.1). The estimate needed here is the following tailored consequence of Burbea’s sharp product inequality [2, Thm. 4.1, p. 257]:

#### Lemma 5.4

Let *q* be analytic in $\mathbb {D}$ and suppose that

$$ q(0)=q'(0)=0, \qquad 1-q\in \mathcal {H}^1(\mathbb {D}), \qquad 1-q(z)\ne 0\quad \text {for all }z\in \mathbb {D}. $$

Then $$q/z^2\in A^2(\mathbb {D})$$,

$$ M:=\left\| 1-q \right\| _{\mathcal {H}^1}\ge 1, $$

and

5.3 $$\begin{aligned} \left\| \frac{q}{z^2} \right\| _{A^2}^2\le (M-1)(M+3). \end{aligned}$$

Moreover, equality holds if and only if q≡0.

#### Proof

Since $\mathbb {D}$ is simply connected and 1-q is zero-free, it has an analytic square root *g*, normalised by g(0)=1. Thus

$$ g^2=1-q. $$

Then $$g\in \mathcal {H}^2(\mathbb {D})$$ and

$$ \left\| g \right\| _{\mathcal {H}^2}^2=\left\| 1-q \right\| _{\mathcal {H}^1}=M. $$

In particular, $$M\ge \left| g(0) \right| ^2=1$$. Since $q^{\prime }\left(0\right)=0$, we have $g^{\prime }\left(0\right)=0$, and hence

$$ g=1+z^2v $$

for some $v\in \mathcal {H}^2(\mathbb {D})$. Parseval’s identity [26, Sec. 3.4, p. 43] gives

$$ \left\| v \right\| _{\mathcal {H}^2}^2=M-1, \qquad \left\| g+1 \right\| _{\mathcal {H}^2}^2=M+3. $$

Moreover,

$$ \frac{q}{z^2}=-(g+1)v. $$

Applying Burbea’s product inequality [2, Thm. 4.1, p. 257] gives

$$ \left\| \frac{q}{z^2} \right\| _{A^2}^2 \le \left\| g+1 \right\| _{\mathcal {H}^2}^2\left\| v \right\| _{\mathcal {H}^2}^2 = (M+3)(M-1). $$

If $v\not \equiv 0$, the equality statement in Burbea’s product inequality [2, Thm. 4.1, p. 257] gives constants $C_1,C_2\in \mathbb {C}\setminus \{0\}$ and $\zeta \in \mathbb {D}$ such that

$$ g(z)+1=\frac{C_1}{1-\overline{\zeta }z}, \qquad v(z)=\frac{C_2}{1-\overline{\zeta }z}. $$

The linear coefficient of g+1 vanishes, while its constant coefficient is equal to 2. Thus ζ=0, so both functions are constant. The identity $g+1=2+z^2v$ then gives v≡0, a contradiction. Consequently, equality holds only if v≡0, which is equivalent to q≡0. The converse is immediately apparent. □

#### Proof of Proposition 5.1

By translation invariance, we may suppose that $0\in \operatorname {int}(E)$. We use the notation of (3.1) and Lemma 5.3 and set

$$ c:={{\,\textrm{cap}\,}}(E), \qquad \eta :=\left\| \frac{q}{z^2} \right\| _{A^2}^2, \qquad M:=\left\| 1-q \right\| _{\mathcal {H}^1}. $$

Since $\left| E \right| =\pi$, equations (5.1) and (5.2) give

$$ \eta =1-c^{-2}, \qquad M=\frac{\mathcal {P}(E)}{2\pi c}. $$

Applying Lemma 5.4, we obtain

$$ \eta \le (M-1)(M+3). $$

Since M≥1, this is equivalent to

$$ M\ge -1+\sqrt{4+\eta }. $$

Consequently,

$$ \frac{\mathcal {P}(E)}{2\pi } =cM \ge c\left( -1+\sqrt{5-c^{-2}}\right) = \sqrt{5c^2-1}-c, $$

which proves the estimate.

If equality holds in the estimate of Proposition 5.1, then equality holds in (5.3). Lemma 5.4 gives q≡0. Hence $\beta _n=0$ for every n≥1, while (5.1) gives c=1. Thus $\Phi (\zeta )=\zeta +\beta _0$, and *E* is a translate of the unit disc. Conversely, a translate of the unit disc gives equality. □

The following logarithmic form is adapted to the variational functional:

#### Lemma 5.5

Let *E* satisfy the assumptions of Proposition 5.1. Then

$$ \mathcal {P}(E)-2\pi \ge 3\pi \log {{\,\textrm{cap}\,}}(E). $$

Moreover, equality holds if and only if *E* is a translate of the unit disc.

#### Proof

Set $c:={{\,\textrm{cap}\,}}(E)$. Lemma 5.2 gives c≥1. In view of Proposition 5.1, it remains to show that

$$ \sqrt{5c^2-1}-c\ge 1+\frac{3}{2}\log c \qquad \text {for }c\ge 1. $$

Define $$F:[1,\infty )\rightarrow \mathbb {R}$$ by

$$ F(c):=\sqrt{5c^2-1}-c-1-\frac{3}{2}\log c. $$

Then F(1)=0 and

$$ F'(c)=\frac{5c}{\sqrt{5c^2-1}}-1-\frac{3}{2c}. $$

Rewriting the numerator gives

$$ F'(c) = \frac{(c-1)(80c^3+20c^2-21c-9)}{2c\sqrt{5c^2-1}\left( 10c^2+(2c+3)\sqrt{5c^2-1}\right) }. $$

The cubic factor is positive on [1,∞) because

$$ 80c^3+20c^2-21c-9 =70+(c-1)(80c^2+100c+79). $$

Thus $F^{\prime }\left(c\right)\geq 0$ for c≥1, with strict inequality for c>1. This proves the estimate.

If $\mathcal {P}(E)-2\pi =3\pi \log {{\,\textrm{cap}\,}}(E)$, then c=1 and $\mathcal {P}(E)=2\pi$. Hence equality also holds in Proposition 5.1, and *E* is a translate of the unit disc. The converse is immediately apparent. □

#### Proposition 5.6

For every $0\le \textrm{We}\le 3$, the unique minimisers of $\mathcal {F}_{\textrm{We}}$ among compact sets whose boundary is given by a rectifiable Jordan curve and area π are unit discs up to translation.

#### Proof

Let *E* be admissible and set $c:={{\,\textrm{cap}\,}}(E)$. By Lemma 5.5,

$$ \mathcal {P}(E)-3\pi \log c\ge 2\pi . $$

Lemma 5.2 gives c≥1. Hence, for $0\le \textrm{We}\le 3$,

$$ \begin{aligned} \mathcal {F}_{\textrm{We}}(E)&=\mathcal {P}(E)-\textrm{We}\pi \log c \\&=\bigl (\mathcal {P}(E)-3\pi \log c\bigr )+(3-\textrm{We})\pi \log c \\&\ge 2\pi . \end{aligned} $$

For the closed unit disc, $\mathcal {P}(\overline{\mathbb {D}})=2\pi$ and ${{\,\textrm{cap}\,}}(\overline{\mathbb {D}})=1$; see [32, Table 5.1]. Thus $\mathcal {F}_{\textrm{We}}(\overline{\mathbb {D}})=2\pi$.

If $\mathcal {F}_{\textrm{We}}(E)=2\pi$ for an admissible set *E*, then $\mathcal {P}(E)-3\pi \log c=2\pi$. Lemma 5.5 implies that $E=x_0+\overline{\mathbb {D}}$ for some $x_0\in \mathbb {R}^2$. This completes the proof. □

### For $\textrm{We}>3$, Elliptic Deformations Lower the Energy

Let us now prove that the threshold is sharp. We consider the one-parameter family of closed ellipses

$$ \mathcal {E}_t:=\{(x_1,x_2)\in \mathbb {R}^2 \; ; \; \textrm{e}^{-2t}x_1^2+\textrm{e}^{2t}x_2^2\le 1\}, \qquad t\in \mathbb {R}. $$

Then $\mathcal {E}_0=\overline{\mathbb {D}}$, and each $\mathcal {E}_t$ is a convex and compact set satisfying that $\left| \mathcal {E}_t \right| =\pi$.

#### Proposition 5.7

For every $\textrm{We}>3$, there exists $\delta _{\textrm{We}}>0$ such that

$$ \mathcal {F}_{\textrm{We}}(\mathcal {E}_t)<\mathcal {F}_{\textrm{We}}(\overline{\mathbb {D}}) \qquad \text {whenever}\qquad 0<\left| t \right| <\delta _{\textrm{We}}. $$

#### Proof

The logarithmic capacity of an ellipse equals half the sum of its semiaxes; see [32, Table 5.1]. We therefore obtain

$$\begin{aligned} \mathcal {I}(\mathcal {E}_t)&=-\log {{\,\textrm{cap}\,}}(\mathcal {E}_t) =-\log \!\left( \frac{\textrm{e}^t+\textrm{e}^{-t}}{2}\right) =-\log (\cosh t) \\&=-\frac{1}{2}t^2+O(t^4), \qquad t\rightarrow 0. \end{aligned}$$

The perimeter of $\mathcal {E}_t$ can be written as

5.4 $$\begin{aligned} \mathcal {P}(\mathcal {E}_t) =\int _0^{2\pi } \sqrt{\textrm{e}^{2t}\sin ^2\theta +\textrm{e}^{-2t}\cos ^2\theta } \textrm{d}\theta , \end{aligned}$$

from which we obtain the expansion

$$ \mathcal {P}(\mathcal {E}_t) =2\pi +\frac{3\pi }{2}t^2+O(t^4), \qquad t\rightarrow 0. $$

Consequently,

$$ \mathcal {F}_{\textrm{We}}(\mathcal {E}_t)-\mathcal {F}_{\textrm{We}}(\overline{\mathbb {D}}) =-\frac{\pi }{2}(\textrm{We}-3)t^2+O_{\textrm{We}}(t^4). $$

For $\textrm{We}>3$, this is negative for all sufficiently small nonzero *t*. □

### Conclusion of the Sharp Threshold

#### Proof of Theorem 1.2

Every planar convex and compact set of positive area has boundary prescribed by a rectifiable Jordan curve. Hence Proposition 5.6 applies to the admissible class in (1.3) and proves the claim for $0\le \textrm{We}\le 3$. For $\textrm{We}>3$, Proposition 5.7 supplies an admissible ellipse with lower energy than the disc. □

#### Remark 5.8

For $\textrm{We}>3$, it remains open to characterise minimisers of (1.3) and, more broadly, those among closures of bounded Jordan domains of area π. It is also an interesting open problem whether, besides the disc and the bifurcation branches constructed in [39] , $\mathcal {F}_{\textrm{We}}$ has critical sets whose boundaries, together with some ψ and λ, solve (1.2). For $\textrm{We}\gg 3$, numerical experiments yield non-circular elongated shapes reminiscent of a stadium.

## Data Availability

Not applicable.
